# Supplementary material for: Mutating chikungunya virus non‐structural protein produces potent live‐attenuated vaccine candidate
Source: EMBO Mol Med. 2019 Apr 23;11(6):e10092. doi: 10.15252/emmm.201810092 (PMC6554673; doi:10.15252/emmm.201810092)
Supplement: Supplementary file 1 — Expanded View Figures PDF [file EMMM-11-e10092-s001.pdf]

Expanded View Figures

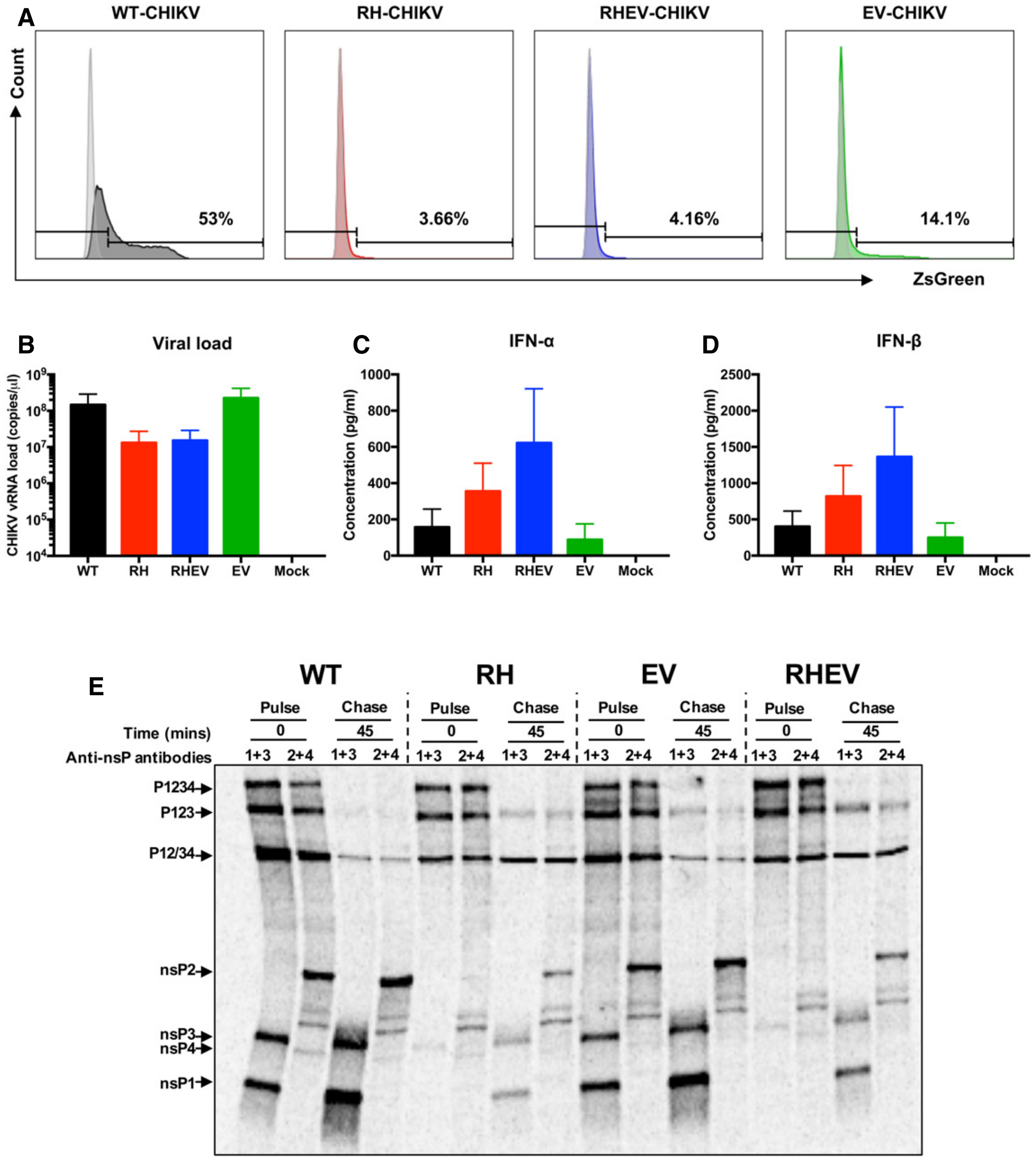

Figure EV1. R532H and E515V mutations in nsP regions limit CHIKV pathogenicity in primary MTFs and affect proteolytic cleavage of nsP1/2.

A Representative fluorescence-activated cell sorting histograms showing CHIKV nsP-mutant infectivity of MTFs at 12 hpi.

B Viral load of MTFs infected with WT-, RH-, RHEV-, and EV-CHIKV (MOI 10, n = 3 per group) harvested at 12 hpi. Error bars are SEM.

C, D Concentrations of soluble (C) IFN-α and (D) IFN-β levels in the supernatant of infected MTFs were measured by type-I interferon detection immunoassay. Data are representative of three independent experiments and are presented as the means ± SEM. Statistical analyses were performed using two-tailed paired t-test.

E BHK-21 cells were infected with WT-CHIKV, RH-CHIKV, EV-CHIKV, or RHEV-CHIKV (MOI 10). At 3 hpi, cells were starved (37°C, 30 min) in methionine- and cysteine-free DMEM. Cells were subsequently pulse-labeled for 15 min with ^{35}S-labeled methionine–cysteine and chased for 45 min in DMEM containing excess unlabeled methionine and cysteine before lysis. NsPs were immunoprecipitated from cell lysates using combination of antibodies directed against nsP1 and nsP3 (1 + 3), or against nsP2 and nsP4 (2 + 4). Immunoprecipitated proteins were denatured by heating in Laemmli buffer and separated using 8% SDS–PAGE and visualized using Typhoon imager.

 © 2019 Agency for Science, Technology and Research (A*STAR)

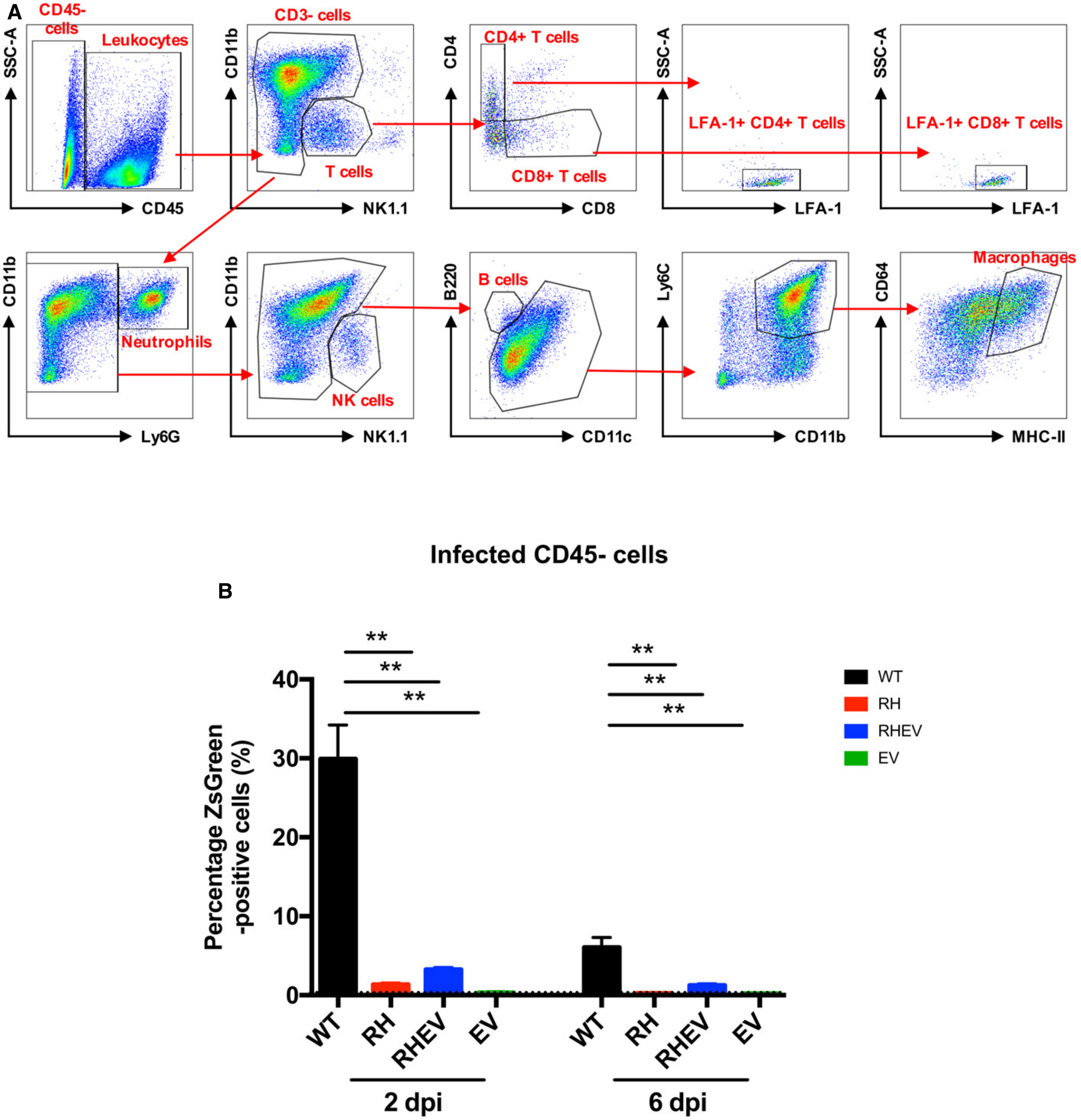

Figure EV2. R532H and E515V mutations in CHIKV nsPs result in lower infectivity at the site of inflammation.

WT C57BL/6 mice were infected subcutaneously with 1E6 PFU ZsGreen-tagged WT-CHIKV, RH-CHIKV, EV-CHIKV, and RHEV-CHIKV at the metatarsal region of the footpad.

A Representative fluorescence-activated cell sorting gating strategy to isolate specific leukocyte subsets.

B Infection at both 2 and 6 dpi was assessed in CD45- cells. Background ZsGreen signals are indicated by the dotted line. The data are presented as the means ± SD (n = 5 per group). Statistical analyses were performed using two-tailed Mann–Whitney U-test (**P = 0.008 RH-CHIKV 2 and 6 dpi, **P = 0.008 RHEV-CHIKV 2 and 6 dpi, **P = 0.008 EV-CHIKV 2 and 6 dpi).

© 2019 Agency for Science, Technology and Research (A*STAR)

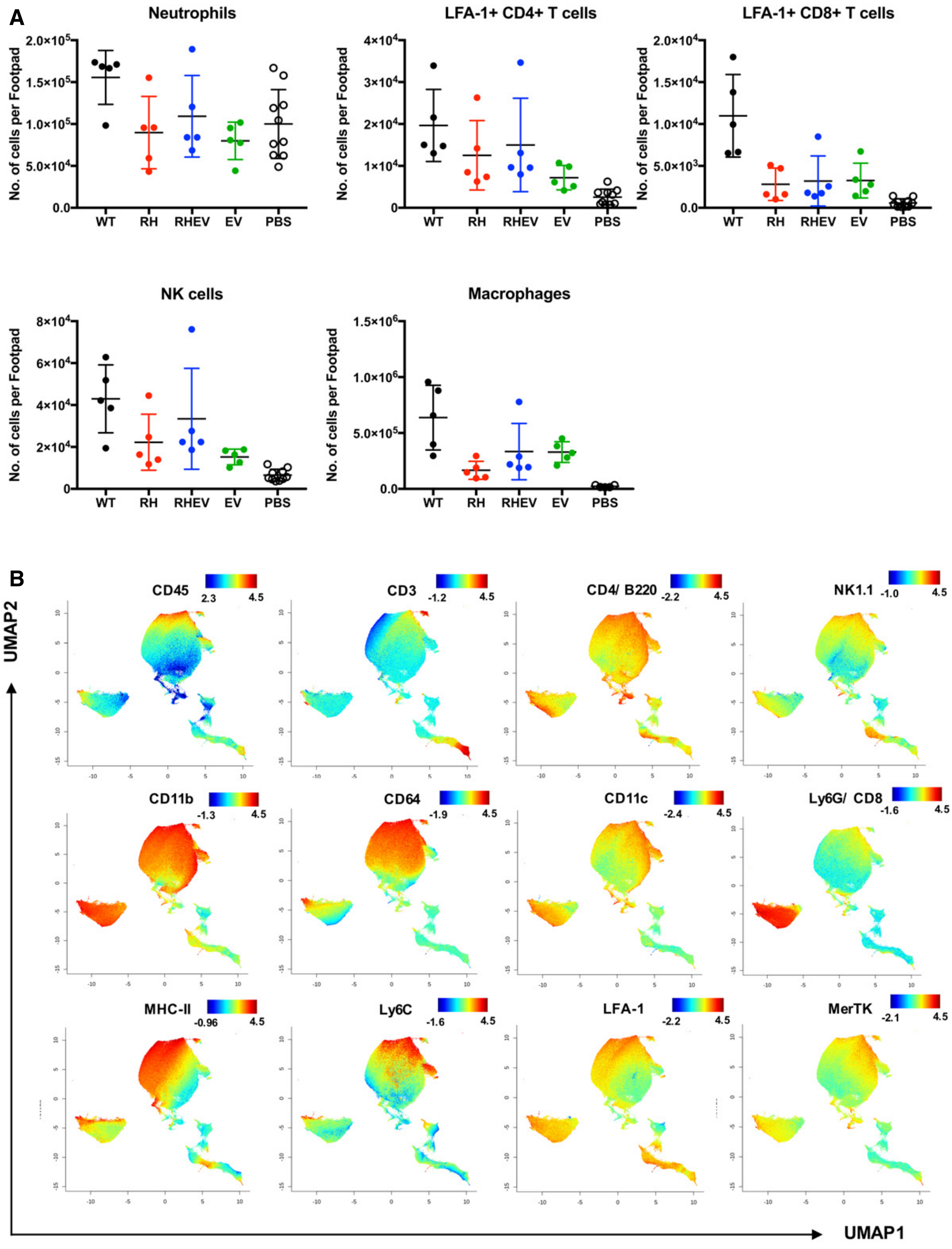

Figure EV3. High-dimensional analysis of fluorescence-activated cell sorting (FACS) data with UMAP dimensionality reduction.

A Absolute numbers of immune subsets present in the joints as determined by immune-phenotyping. Data are presented as the means ± SD (n = 5 per group, except PBS n = 10). No significance was obtained between the groups by Kruskal–Wallis test with Dunn's multiple comparisons.

B UMAP dimensionality reduction color-coded by marker intensity. Representative visualizations of key surface marker expression levels on UMAP transformed FACS data. Joint cells were single-stained for surface markers and acquired by flow cytometry.

 © 2019 Agency for Science, Technology and Research (A*STAR)

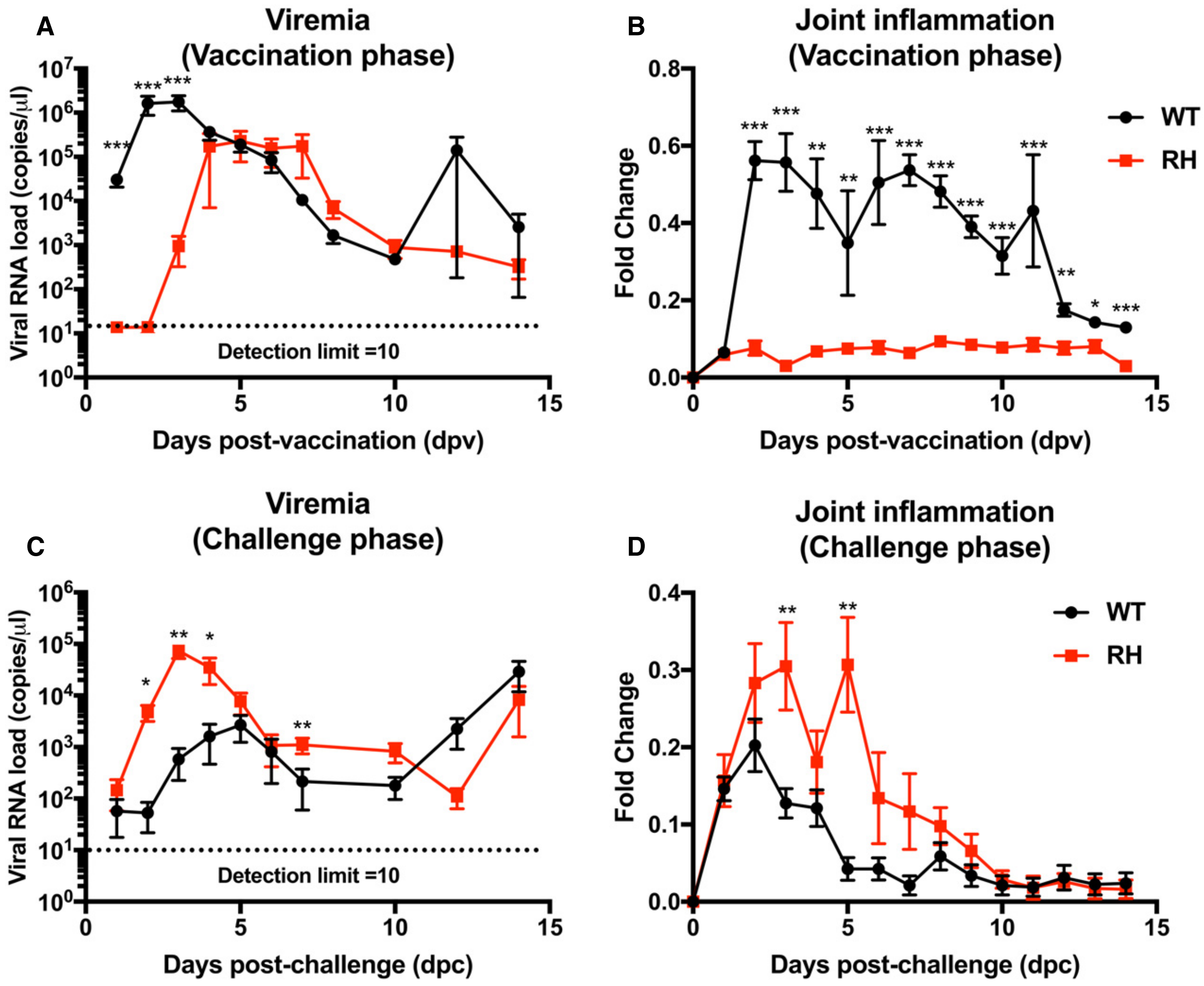

Figure EV4. Antibodies are important in the protection against WT-CHIV challenge.

μMT mice were vaccinated with either 1E6 PFU WT-CHIKV or RH-CHIKV (n = 6–8 per group) at the metatarsal region of the footpad.

A, B (A) Viremia and (B) joint inflammation in vaccinated mice were monitored over 2 weeks. Viremia is detected with CHIKV nsP1 probe via qRT–PCR. Statistical analyses were performed using two-tailed Mann–Whitney *U*-test; viremia (***P = 0.000666 1–3 dpv) and joint inflammation (***P = 0.000333 2 dpv, ***P = 0.000666 3 dpv, **P = 0.006 4 dpv, **P = 0.005 5 dpv, ***P = 0.000666 6 dpv, ***P = 0.000333 7–8 dpv, ***P = 0.000666 9–10 dpv, ***P = 0.000333 11 dpv, **P = 0.001 12 dpv, *P = 0.028 13 dpv, ***P = 0.000666 14 dpv). Error bars are in SEM.

C, D (C) Vaccinated μMT mice were challenged with 1E6 PFU WT-CHIKV (n = 8–10 per group) at the metatarsal region of the footpad. Joint inflammation and (D) viremia progression in challenged μMT mice were monitored over 2 weeks. The data are presented as the means ± SEM and are representative of two independent experiments. Statistical analyses were performed using two-tailed Mann–Whitney *U*-test; viremia (*P = 0.016 2 dpc, **P = 0.009 3 dpc, *P = 0.016 4 dpc, **P = 0.009 7 dpc) and joint inflammation (**P = 0.006 3 dpc, **P = 0.001 5 dpc).

© 2019 Agency for Science, Technology and Research (A*STAR)

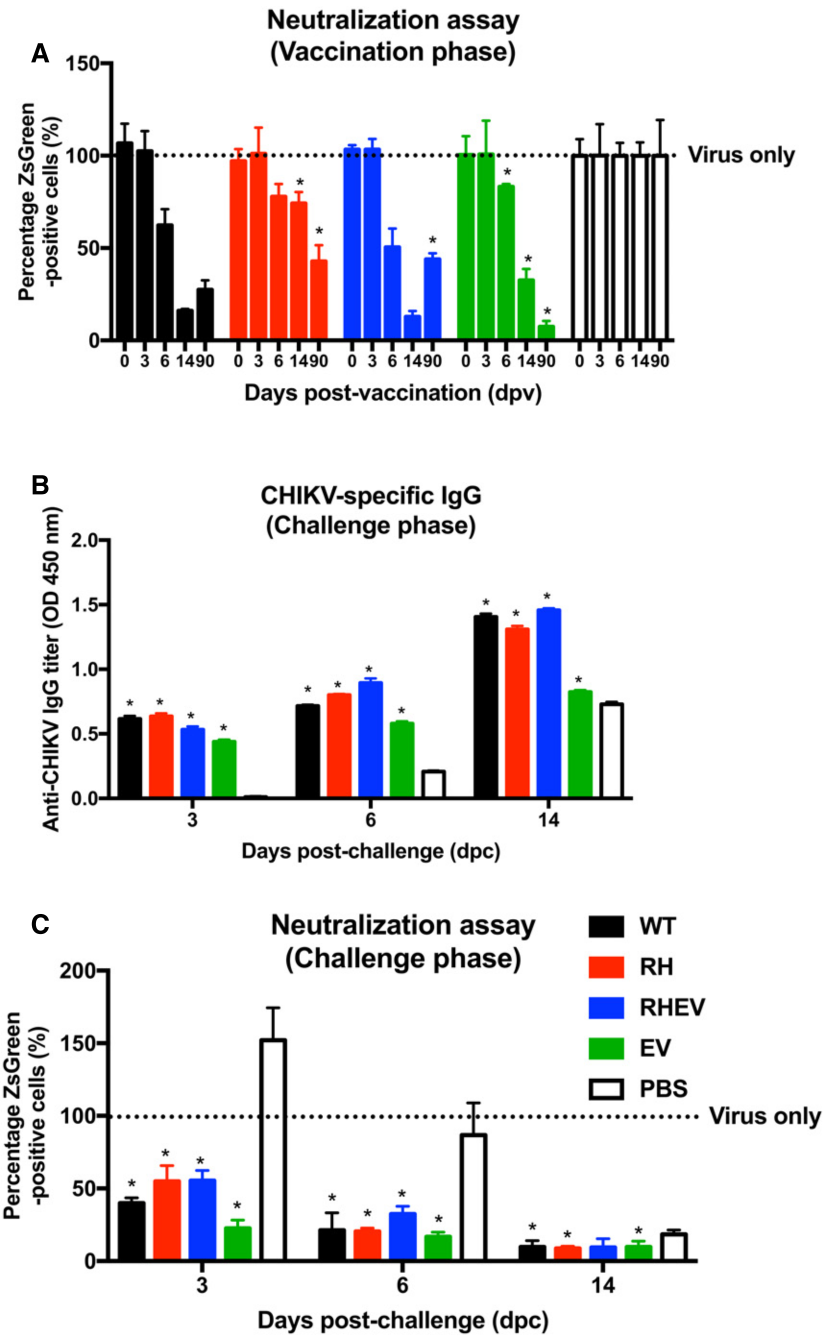

Figure EV5. Vaccination with attenuated CHIKV induces a robust antibody response against WT-CHIKV challenge.

A The neutralizing capacity of the pooled sera was characterized at 1:1,000 dilution in HEK293T cells infected with WT-CHIKV (MOI 5). The percentage infection was normalized to sera isolated from mock-vaccinated mice. Tested sera were obtained at 0, 3, 6, 14, and 90 dpv. Statistical analyses were performed using two-tailed Mann–Whitney *U*-test against the positive control (WT-CHIKV) on the respective dpv (**P* = 0.029 RH-CHIKV 14 and 90 dpv, **P* = 0.029 RHEV-CHIKV 90 dpv, **P* = 0.029 EV-CHIKV 6, 14 and 90 dpv). Error bars are in SD, from 4 replicates.

B Anti-CHIKV IgG titers were determined with a CHIKV virion-based ELISA. Sera from four animals were pooled and diluted 1:500 for the assay. Statistical analyses were performed using two-tailed Mann–Whitney *U*-test against the mock-vaccinated group on each respective dpc (**P* = 0.029 RH-CHIKV 3, 6 and 14 dpc, **P* = 0.029 RHEV-CHIKV 3, 6 and 14 dpc, **P* = 0.029 EV-CHIKV 3, 6 and 14 dpc). Error bars are in SD, from 4 replicates.

C Neutralizing capacity of the pooled sera was characterized at a 1:1,000 dilution in HEK293T cells infected with WT-CHIKV (MOI 5). Data are presented as the means ± SD. The percentage infection was normalized to virus-only infection. Sera tested were obtained at 3, 6, and 14 dpc. Statistical analyses were performed using two-tailed Mann–Whitney *U*-test against the mock-vaccinated group on each respective dpc (**P* = 0.029 RH-CHIKV 3, 6, and 14 dpc, **P* = 0.029 RHEV-CHIKV 3 and 6 dpc, **P* = 0.029 EV-CHIKV 3, 6, and 14 dpc). Data is representative of 4 replicates.

© 2019 Agency for Science, Technology and Research (A*STAR)
